# Impact of Fructose-Enhanced Solid and Soft Drink Diets on Metabolism, Physiology, and Gut Microbiome in Pregnant Rats

**DOI:** 10.1155/bmri/6902453

**Published:** 2025-08-13

**Authors:** Xiaoyuan Han, Linda D. Shortliffe, Neeraja Kambham

**Affiliations:** ^1^Department of Biomedical Sciences, University of the Pacific, Arthur A. Dugoni School of Dentistry, San Francisco, California, USA; ^2^Department of Urology, Stanford University School of Medicine, Palo Alto, California, USA; ^3^Department of Pathology, Stanford University School of Medicine, Stanford, California, USA

**Keywords:** dyslipidemia, fructose, gestational diabetes, gut microbiome, pregnancy, soft drink consumption

## Abstract

**Introduction:** High fructose consumption is linked to hypertension and metabolic disorders. We hypothesize fructose (solid and/or liquid) may further influence dietary patterns. To investigate potential effects, we compared eating behaviors, physiological and metabolic measures, and gut microbiome in pregnant rats fed solid or liquid fructose-enhanced (soft drink) diets to those fed standard chow.

**Methods:** Pregnant rats were assigned to three dietary groups: (1) standard chow and water (CW), (2) standard chow and decarbonated soft drink (CS, decarbonated Sprite with 5.89% fructose *w*/*v*), and (3) 60% fructose chow and water (FW). Body weight, blood pressure, food and liquid intake, fasting blood glucose, plasma insulin, and lipid profiles were measured weekly. Fecal samples were collected before and during pregnancy. At euthanasia, livers and kidneys were analyzed for pathological changes.

**Results:** Data from five rats per group were analyzed. The CS group showed nearly a twofold increase in fluid intake and urinary output at midpregnancy. Both solid and liquid fructose groups had increased carbohydrate calorie intake per kilogram of body weight, while protein and fat intake decreased. Although all rats showed pregnancy-related dyslipidemia, the FW group experienced a more pronounced increase. Pregnancy and the FW diet also led to distinct shifts in the fecal gut microbiome.

**Conclusions:** This pregnant rat model highlights the impact of varying fructose diets during physiological stress. Pregnant rats on high-fructose diets (CS, FW) increased their carbohydrate intake at the expense of protein and fat intake when compared with pregnant rats on a routine diet. CS rats showed a marked increase in soft drink intake and urine. These findings have potential nutritional and health implications for pregnancy and long-term health and warrant further investigation and confirmation.

## 1. Introduction

In recent decades, carbohydrate consumption has increased, largely due to the widespread use of processed high-fructose corn syrup (HFCS). Elevated intake of fructose in both humans and animals has been associated with conditions such as hypertension, diabetes, and metabolic syndrome [1–3] with variable outcomes in males and females. While some research examines how changes in carbohydrate intake during pregnancy affect offspring, less attention has been given to the effects on the mother's pregnancy and her long-term health [4–11]. In our previous research, we explored the effects of fructose on pregnancy using an animal model, as such studies are difficult or impossible to conduct in humans [12]. We found that pregnancy itself creates a physiological stress condition that can accentuate and accelerate the effects of fructose dietary changes, revealing outcomes that might otherwise take prolonged observation [12, 13].

In preliminary studies, we observed that various carbohydrates (solid vs. liquid) may alter dietary intake patterns. Specifically, rats appear to prefer soft drink hydration over water. Based on this observation and literature associating soft drinks with metabolic syndrome and other adverse health outcomes in humans, we compared eating patterns, physiological and metabolic measures, and gut microbiome of pregnant rats consuming fructose-enhanced chow and “soft drink.”

## 2. Methods

### 2.1. Study Protocol

This study was approved by the Administrative Panel on Laboratory Animal Care (APLAC), Department of Laboratory and Animal Medicine Veterinary Services of Stanford University Institutional Review Board, and followed guidelines from the American Association for the Accreditation of Laboratory Animal Care. Ten-week-old female Sprague Dawley rats (250 g, Charles River Laboratories) were acclimated for 1 week on a standard chow diet with ad libitum water (Rodent Diet, Teklad global), maintained on a 12-h light/dark cycle, and then bred. Pregnancy (Day1) was confirmed by detecting a copulation plug in the vagina or sperm in vaginal cytology [14, 15].

**Figure 1:**
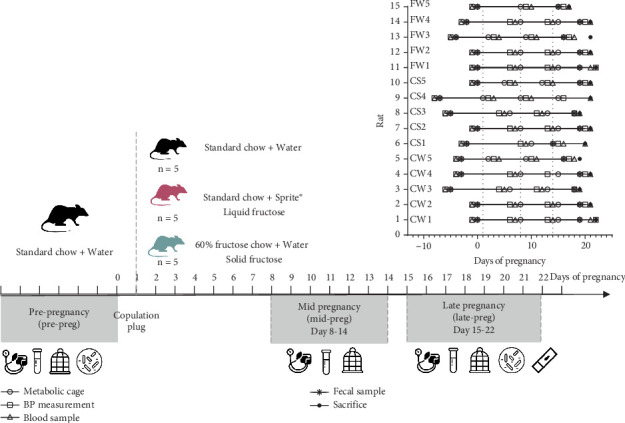
Diagram of the experiment to monitor solid and liquid fructose and standard diets on metabolism, physiology, and gut microbiome during pregnancy. The analyzed 15 rats were distributed among three dietary groups with gestational days numbered after the appearance of a copulation plug. Sample collection times (metabolic cage [circle], BP [square], blood [triangle], and feces [star] or sacrifice time [dot]) are indicated. Data are compared among prepregnancy (pre-preg), middle of pregnancy (mid-preg, Days 8–14), and late pregnancy (late-preg, Days 15–22).

Pregnant rats were randomly assigned to one of three dietary groups: (1) standard chow (Teklad global 18% protein rodent diet, Teklad Custom Research Diet, Harlan Laboratories, Madison, Wisconsin) and water ad libitum (CW), (2) standard chow and decarbonated soft drink ad libitum (Sprite, 5.89% fructose) (CS), and (3) 60% fructose chow (TD.89247, Teklad Custom Research Diet, Harlan Laboratories, Madison, Wisconsin) and water (FW). All diets were provided ad libitum (24 h a day, 7 days a week, Figure 1).

Blood pressure (BP), blood samples, and 24-h metabolic cage measurements were taken weekly from prepregnancy (pre-preg) through pregnancy until euthanasia, occurring 1–3 days before parturition (median: Day 21, range: Days 17–22). At euthanasia, blood was collected, fetal number and total weight recorded, and major organs (heart, liver, pancreas, kidneys, fat, and bladder) weighed and preserved in 10% buffered formalin. Weekly testing sequences varied slightly due to conception timing and metabolic cage availability [14] (Figure 1).

### 2.2. BP

Rats were trained for BP measurement during the 1-week acclimation. Weekly, after overnight fasting and a 2–4 h quiet period, systolic BP (mmHg) was recorded as the mean of 15 consecutive readings using a noninvasive tail-cuff system (BP-2000, BP analysis system, Visitech Systems Inc., North Carolina, United States), as previously described [16, 17].

### 2.3. Blood Analyses

After overnight fast, blood samples were collected weekly by retro-orbital puncture and at euthanasia via cardiac puncture. Blood glucose was measured with a Bayer Contour glucose meter. Serum samples were stored in 55-*μ*L aliquots at −80°C for further analysis, including insulin (rat ultrasensitive insulin ELISA, ALPCO Diagnostics), triglycerides (TG) (enzymatic colorimetric assay, Cayman Chemical), HDL, LDL/VLDL, and total cholesterol (EnzyChrom™ AF HDL/LDL/VLDL Assay Kit, BioAssay Systems), and creatinine (QuantiChrom™ Creatinine Assay Kit [DICT-500], BioAssay Systems). Insulin resistance was calculated using HOMA-IR [18]:

(1) $$\begin{array}{c} \text{HOMA}‐\text{IR}=\frac{\text{fasting glucose }\left(\text{mM}/\text{L}\right)\times \text{fasting insulin }\left(\text{mU}/\text{mL}\right)}{22.5}. \end{array}$$

### 2.4. Metabolic Cage Monitoring

Each rat was housed individually in Tecniplast metabolic cages for 24-h periods each week. Data collected included body weight (BW), chow and liquid intake, and fecal and urinary output.

Fresh fecal pellets were snap-frozen and stored at −80°C for DNA extraction. Caloric intake was calculated using dietary composition data (Table S1). To avoid stress, metabolic cage monitoring and BP measurements were conducted at least 3 days apart.

### 2.5. Histopathologic Examination of Liver and Kidney

Liver and kidney tissues were paraffin-embedded, sectioned to 5 *μ*m, and stained with hematoxylin–eosin for the liver (HE, American MasterTech Science Inc.) and Periodic acid-Schiff for the kidney (PAS, American MasterTech Science Inc.). Tissues were randomized, deidentified, and examined by a pathologist (N.K.). Liver histopathology was assessed for steatosis, ballooning, and fibrosis, while kidney pathology was evaluated for glomerulosclerosis, tubulointerstitial fibrosis, and inflammation as previously described [12, 19].

PAS-stained kidney sections were also analyzed using ImageJ software [20]. For each rat, three cortical areas were imaged by Leica Light Microscopy at 200× magnification. Glomerular area was measured for the two longest cross-sectional glomeruli in each image, totaling six glomeruli per rat. Glomerular volume (GV) was calculated using the Weibel and Gomez formula [21]:

(2) $$\begin{array}{c} \text{GV}={\left(\text{glomerular area}\right)}^{1.5}\times \frac{1.38}{1.01}\\ \left(1.38:\text{shape coefficient};1.01:\text{size distribution coefficient}\right). \end{array}$$

Tubular diameter (TD) was determined from the circular cross-sections of five renal tubules per image (15 measurements per rat), as described previously [20, 22].

### 2.6. Fecal Microbiome Analysis

Fecal samples were collected pre-preg and late pregnancy (late-preg) (median: Day 19, range: Days 15–19) (Figure 1). Fecal DNA was extracted using a Qiagen fecal DNA extraction kit (Qiagen) and sequenced by Sierra Genomics (San Mateo, California) via 16S rRNA gene sequencing on an Illumina MiSeq platform. The V4 hypervariable region was analyzed using Mothur (https://www.mothur.org) to generate “operational taxonomic units” (OTUs), and the R vegan package was used for statistical analysis (https://cran.r-project.org/web/packages/vegan/index.html).

### 2.7. Statistical Analysis

Data were analyzed using GraphPad Prism 9 and R (Version 4.1.2) with results presented as mean ± standard deviation (SD). *p* values less than 0.05 were considered significant.

Two-way ANOVA (diet × time) with Tukey's post hoc test was used to compare measures across different timepoints: pre-preg, midpregnancy (mid-preg, Days 8–14), and late-preg (Days 15–22). If multiple values were taken, the closest to Day 11 mid-preg (Days 8–14) and Day 22 (late-preg) was used.

One-way ANOVA with Bonferroni post hoc test was used for organ weight and pathology.

Paired *t*-tests compared microbial abundance (OTUs) pre-preg versus late-preg, and Student's *t*-tests compared microbiome differences between diet groups at the same time point.

## 3. Results

### 3.1. Study Animals

Twenty-one rats were randomized into three dietary groups. Due to unforeseen procedural issues, two rats from each group were excluded from analyses. The final data set included 15 rats (CW = 5, CS = 5, and FW = 5, Figure 1). Missing data from specific time points occurred due to the following reasons: (1) FW5 died during anesthesia on pregnancy Day 17, preventing collection of BP, blood, and metabolic cage data beyond this point; Day 17 blood and tissue specimens were used as final data points; (2) CS1 did not complete metabolic cage and other measures during pregnancy Week 1; (3) CW4 experienced a failed retro-orbital blood collection on Day14; and (4) FW4 lacked sufficient blood for lipid analysis. Despite these exclusions, all rats had terminal blood and pathological specimens available.

A total of 58 data sets are complete for BP and metabolic cage measurements (15 pre-preg and 43 during pregnancy: CW: 15; CS: 14; FW: 14). Similarly, 58 blood specimens were collected, with 57 values available for HDL/VLDL/LDL analyses due to missing data from FW4. All microbiome and pathologic specimens were analyzed.

Study numbers for the fewest number of animals in each group to obtain a 20% change in fructose fluid intake based on past studies, including 20% animal loss during pregnancy, were calculated. Unanticipated breeding (timed pregnancy) issues occurred, altering some timing.

### 3.2. Fructose Soft Drink Increases Liquid and Carbohydrate Intake

**Figure 2:**
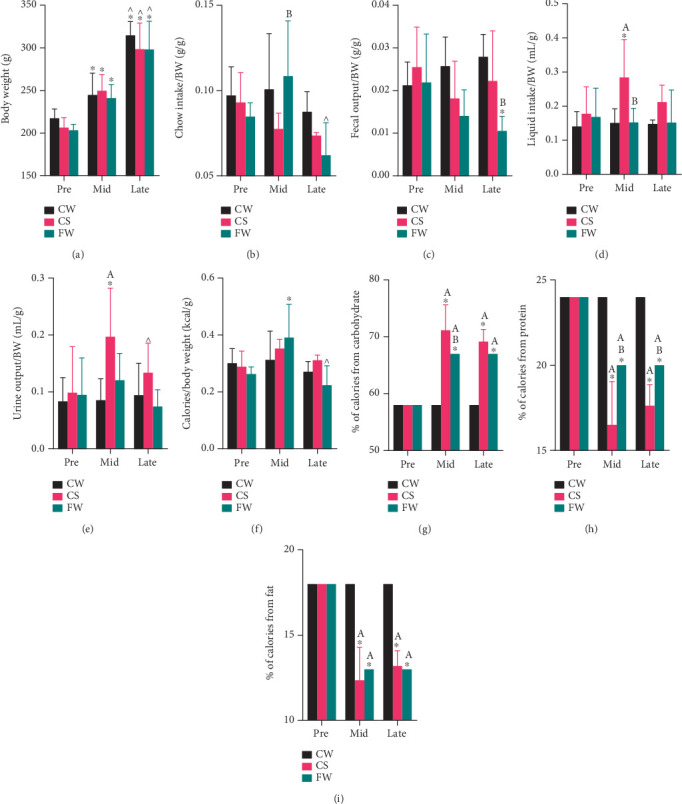
Physiological measures and macronutrient intake of pregnant rats eating standard chow and water (CW, black), standard chow and Sprite (CS, liquid fructose, red), and 60% fructose chow and water (FW, solid fructose, green) diets and monitored in metabolic cages for 24 h. Bar graphs compare (a) body weight, (b) chow intake, (c) fecal output, (d) liquid intake, (e) urinary output, and macronutrient intake ((f) total calorie intake, (g) carbohydrates, (h) protein, (i) fat) across three time points: prepregnancy (Pre), midpregnancy (Mid, Gestational Days 8–14), and late pregnancy (Late, Gestational days 15–22). Fructose-containing diets are associated with higher carbohydrate intake and decreased fat and protein. CS shows higher drink and urine volumes than other diets. Statistical differences are analyzed using two-way ANOVA followed by Tukey's post hoc test. *p* < 0.05 was indicated by ∗ (vs. prepregnancy in the respective dietary group), ^ (vs. midpregnancy in the respective dietary group), A (vs. CW at the respective timepoint), or B (vs. CS at the respective timepoint).

BW increased significantly during mid-preg and late-preg, with no differences between dietary groups (Figure 2a and Figure S1E). When normalized to BW (divided by BW), chow intake was higher in FW than in CS at mid-preg and decreased in FW during late-preg (Figure 2b and Figure S1A). FW exhibited reduced fecal output during late-preg compared to both their pre-preg levels and CW at the same stage (Figure 2c and Figure S1B).

CS showed a nearly twofold increase in both liquid intake (Table S2, 0.284 ± 0.111 vs. 0.178 ± 0.079 mL/g) and urinary (Table S2, 0.197 ± 0.086 vs. 0.099 ± 0.080 mL/g) output at mid-preg, which returned to pre-preg levels by late-preg (Figure 2d,e and Figure S1C&D).

Total caloric intake (normalized to BW), calculated from chow and liquid intake, was elevated in FW at mid-preg and then, at late-preg, dropped toward pre-preg levels (Figure 2f, Figure S2A). While only FW showed an increase in total caloric intake, both fructose diet groups had a higher proportion of carbohydrate-derived calories during pregnancy (Figure 2g and Table S2, 71.1 ± 4.5 in FW and 69.2 ± 2.1 in CS vs. 58.0% ± 0.0% in CW) and a concurrent reduction in protein (Figure 2h and Figure S2D) and fat intake (Figure 2i and Figure S2C).

### 3.3. Fructose Impacts Blood Glucose, Insulin, and Lipid Profile

**Figure 3:**
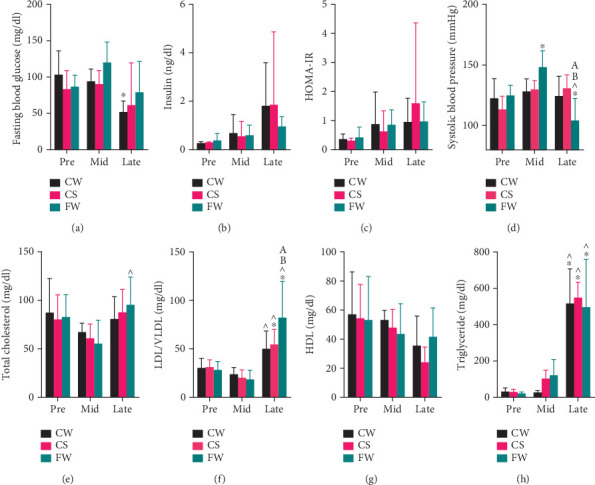
Serum metabolic parameters and systolic blood pressure measures in pregnant rats eating standard chow and water (CW, black), standard chow and Sprite (CS, liquid fructose, red), and 60% fructose chow and water (FW, solid fructose, green) diets. Bar graphs compare (a) fasting blood glucose, (b) insulin, (c) HOMA-IR, (d) systolic blood pressure, (e) total cholesterol, (f) LDL/VLDL cholesterol, (g) HDL cholesterol, and (h) triglyceride for three time points: prepregnancy (Pre), midpregnancy (Mid, Gestational Days 8–14), and late pregnancy (Late, Gestational Days 15–22). While only FW shows elevated BP at midpregnancy, all groups show the increased triglyceridemia of pregnancy. Statistical differences are analyzed using two-way ANOVA followed by Tukey's post hoc test. *p* < 0.05 was indicated by ∗ (vs. prepregnancy in the respective dietary group), ^ (vs. midpregnancy in the respective dietary group), A (vs. CW at the respective timepoint), or B (vs. CS at the respective timepoint).

Fasting blood glucose (FBG) decreased significantly in CW during late-preg compared to pre-preg (Figure 3a, Table S2, and Figure S3A). Insulin and HOMA-IR increased in all groups during pregnancy (Figure 3b,c and Figure S3B&C).

While lipid analysis revealed that LDL/VLDL and TG levels increased significantly in all groups during late-preg (Figures 3f, 3g, and 3h, Table S2, and Figure S3F&G), FW exhibited a significantly higher LDL/VLDL ratio at late-preg than either CW or CS (Figure 3f and Table S2, 82.3 ± 37.4 mg/dL). In FW, total cholesterol significantly increased in late-preg compared to mid-preg (Figure 3e and Figure S3D). No significant changes in HDL were observed across the three dietary groups throughout pregnancy (Figure 3h and Figure S3E).

### 3.4. Fructose Alters BP

Systolic BP in FW increased at mid-preg but returned to baseline by late-preg (Figure 3d, Table S2, and Figure S3H). No significant BP changes were observed in CS.

### 3.5. Fructose Diets and Pregnancy Alter Gut Microbiome

**Figure 4:**
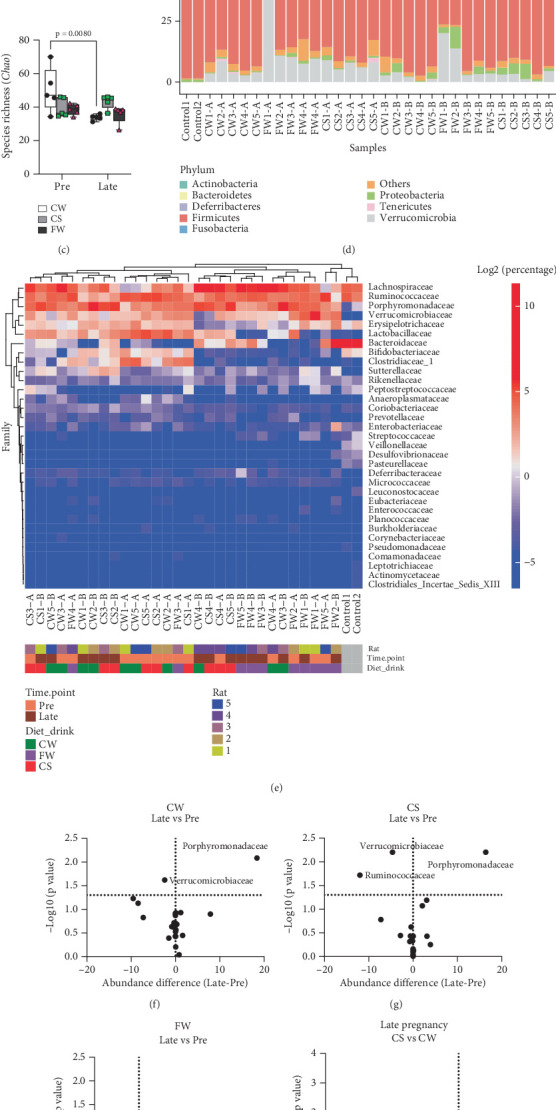
Rat gut microbiome findings prepregnancy (Pre) and late pregnancy (Late, Gestational Days 15–22) for three diets (CW: standard chow and water; CS: standard chow and Sprite, liquid fructose; FW: 60% fructose chow and water, solid fructose). Nonmetric multidimensional scaling (NMDS) plots illustrate gut microbiome shifts associated with (a) pregnancy and (b) diets. (c) Boxplot depicts changes in species richness (Chao1 index), analyzed by two-way ANOVA with repeated measures following Bonferroni post hoc test. (d) Bar chart displays gut microbiome composition at the phylum level. (e) Heatmap (family level) reveals distinct clustering of late-pregnancy and FW samples compared to prepregnancy and other diet groups. (f–h) Volcano plots highlight microbial families with altered relative abundance (OTUs) at late pregnancy in CW and CS but not FW rats (Student's *t*-test). (i–k) Volcano plots reveal distinct microbial family changes associated with FW and CS diets at late pregnancy (Student's *t*-test). Porphyromonadaceae and Lactobacillaceae abundance are lower at late pregnancy compared to CW and CS.

Pregnancy and FW diet induced distinct shifts in the fecal gut microbiome (Figure 4a,b). The *α*-diversity or species richness, as measured by the Chao1 index [23], decreased at late-preg in CW (Figure 4c). At the phylum level, *Firmicutes* and *Bacteroidetes* remained dominant in all groups (Figure 4d). Heatmap analysis at the family level shows clear clustering of late-preg and FW samples, distinguishing them from pre-preg and other dietary groups (Figure 4e).

Analysis of bacterial family changes (Figures 4, 4, 4, 4, 4, and 4) revealed that CW and CS have increased relative abundance (OTUs) of *Porphyromonadaceae* and decreased *Verrucomicrobiaceae* abundance at late-preg (Figure 4; CW, late-preg vs. pre-preg: 28.76% ± 10.63% vs. 10.36% ± 4.76%, *p* = 8.24e − 3; 2.01% ± 1.51% vs. 4.44% ± 2.41%, *p* = 2.40e − 2; CS: 29.83% ± 3.37% vs. 13.43% ± 3.89%, *p* = 6.26e − 3; 2.54 ± 1.63 vs. 7.19 ± 1.64; *p* = 6.25e − 3). FW exhibited lower *Porphyromonadaceae* abundance at late-preg compared to CW and CS (Figure 4, FW vs. CW: 8.81% ± 5.43% vs. 28.76% ± 10.63%, *p* = 9.77e − 3; FW vs. CS: 8.81% ± 5.43% vs. 29.83% ± 3.37%, *p* = 1.95e − 4). FW also had lower *Lactobacillaceae* abundance at late-preg (Figure 4; FW vs. CW: 0.02% ± 0.01% vs. 4.83% ± 3.31%, *p* = 3.14e − 2; FW vs. CS: 0.02% ± 0.01% vs. 6.33% ± 2.49%, *p* = 4.75e − 3).

### 3.6. Pathology

No significant differences were found in organ weights, fetal number, or fetal weight (Table S3). FW livers showed signs of microsteatosis, but no other kidney or liver histopathological changes were detected (Table S3 and Figure S4).

## 4. Discussion

The literature on physiologic and metabolic effects of high-fructose diets is complex and confusing. Reports of high-fructose dietary effects differ from comparisons using different species, sex, and fructose concentrations, compositions, and sources. Human studies report estimated fructose content of solid and/or liquid, and animal experiments may use high-fructose chow (usually 65% fructose enhanced) or fructose solutions (10%–20% *w*/*v*) or soft drinks (5.89% fructose *w*/*v*). Although animal studies enable restrictive fructose diets, results remain confounded by animal species, sex, concentration, form (solid vs. liquid) of fructose diet, and conditions.

Even though high dietary fructose-associated hypertension has been written about extensively [24], results show that fructose effects are sex-dependent [25, 26]. Male rats fed a high-fructose diet develop hypertension, whereas female rats do not, unless ovariectomized. Proposed causes for sex-based response differences are complex and attributed to intestinal and renal salt handling [27], insulin resistance, leptin responses [26], and differential renal cortical protein expression resulting in different sex-related renal tubular responses [28]. As a result, some investigators study only male rats, precluding variable responses and comparison to females.

Our studies focus upon the female response to a high-fructose diet in pregnancy and how fructose may change dietary patterns and associated physiologic and metabolic measures in this altered physiologic state. As normal pregnancy accelerates many physiologic and metabolic processes and this represents a state of renovascular stress [12, 13], we hypothesized that pregnancy stress may accentuate responses to diet that might not be observed otherwise.

With concerning reports that high fructose and soft drink intake, specifically, are associated with metabolic syndrome and other detrimental physiologic effects [24, 29–32], we questioned whether the pregnancy stress model might reveal how HFCS-containing foods alter food consumption patterns and associated physiologic and metabolic measures during an important physiologic time. Since humans typically do not consume a 60% fructose diet but soft drinks are frequently consumed and are proposed to affect pregnancy adversely, we observed and compared dietary patterns of pregnant rats on ad libitum soft drink, high-fructose chow, and rat routine diet. We selected decarbonized Sprite because it is a popular commercially available soft drink containing HFCS, and we wished to avoid caffeine present in other popular drinks [33]. Sprite contains 5.89% fructose, which is calculated from the composition of HFCS (55% fructose and 45% glucose).

Our previous studies showed that pregnant rats fed a high-fructose diet developed mid-preg hypertension and elevated TG with corresponding insidious liver and kidney histopathologic signs, whereas the nonpregnant rats did not. Findings of mid-preg hypertension in FW are confirmed in this study [12]. In comparison, CS rats did not exhibit BP elevation. It is notable that this differs from findings of Monteiro et al., who reported elevated BP in both nonpregnant and pregnant rats consuming high-fructose fluid (20% fructose solution) at 28 days post delivery [34]. These investigators used a 20% D-fructose solution that differs from soft drinks containing HFCS and has important composition differences from the commercial soft drink used in our study and measured BP at a different time [33].

Although we used CS to examine soft drinks, we used FW for comparison with previous findings by us and other groups [12, 35]. We confirmed high fructose associated increased BP and dyslipidemia during pregnancy, but we failed to confirm other earlier findings in our FW comparison group. FW shows only trends without statistical significance toward elevated FBG, insulin resistance, and HOMA-IR that we and others showed [35]. Moreover, despite elevated TG in FW, histological analysis of liver tissue did not show clear steatosis (Table S3), nor renotubular damage and interstitial inflammation as we and others reported [12, 36, 37]. This is likely due to protocol variations, such as small sample size, shorter diet duration, and earlier timing of animal sacrifice. While it is likely that metabolic changes precede histopathologic ones, this timing is unclear.

We found that soft drink (Sprite) changed the hydration and urinary pattern. Confirmed using metabolic cage studies, CS increased fluid intake and urinary output (Figure 2), whereas FW showed no increases. To our knowledge, a “soft drink” associated increase in fluid and urine during pregnancy has not been noted by others. While Sharma et al. reported both male and female mice fed 65% fructose chow for 3 months had an increase in urinary volume with females having a greater volume, these studies were performed on male and female mice fed for a longer duration (3 months vs. our 21 days) and were unable to confirm BP elevation in either sex, and females were not pregnant. While they propose that males develop proximal and females more distal tubular dysfunction in response to fructose feeding, they also found no renal glomerulopathy and attribute the lack of hypertension to complex strain-specific findings [28].

In our measures, no groups showed significant serum creatinine differences; measures of late creatinine in FS were mostly below the level of assay detection (< 0.1 mg%), and these results need confirmation (Table S2). These observations may be mediated by complex fructose intestinal absorption and/or renal distal tubular dysfunctional changes causing osmotic diuresis or other renal dysfunctional changes leading to increased urinary output. Further discussion of mechanisms would be conjectural, as the previously referenced studies do not apply to changes of pregnancy [28]. This study was not designed to determine the underlying differences between pregnancy and specific aspects of renal metabolism.

In either fructose-enhanced diet (CS, FW), rats consumed more of the fructose-containing food than rats on the routine diet (CW) resulting in both FW and CS having higher carbohydrate intake than CW. When fructose is part of hydration, CS increased soft drink and, consequently, carbohydrate intake and decreased chow intake, thus decreasing protein and fat consumption. When fructose is in the chow, FW initially increased and then decreased chow intake, thereby increasing carbohydrate intake but decreasing protein and fat as fixed components of the chow (Figure 2g and Figure S2B). Regardless of diet, however, the three pregnant rat groups gained weight at similar rates and consumed similar total calories, yet proportional dietary carbohydrate, protein, and fat diets differ depending on their eating pattern (Figure 2). Both FW and CS had a higher percentage of carbohydrates in their total caloric intake—67% ± 0% for FW and 71.1% ± 4.5% for CS—compared to those on the standard chow diet (58% carbohydrate). Notably, the CS group that had the highest carbohydrate intake consumed the least chow, resulting in a significant decrease in protein and fat.

The gut microbiome is influenced by diet and physiological state [38, 39] and high fructose consumption has been associated with microbiome alterations [40]. *Lactobacillaceae* abundance is associated with immune system modulation and gut health [41], and studies link decreases in *Lactobacillus spp.* to states of dysbiosis [42]. In our study, we observed changes in the abundance of the *Porphyromonadaceae* family of bacteria, a key component of the human gut microbiome [43]. While CW rats showed an increase in *Porphyromonadaceae* at late-preg, at a similar time, FW had reduced abundance of both *Porphyromonadaceae* and *Lactobacillaceae* compared with CW and CS (Figure 4). While our CS group did not show reduced abundance, others measured late-preg microbiome changes from drinking a 10% fructose solution [39]. Song et al. found microbiome changes on a 10% solution and not on male rats drinking a 3% solution, thus most likely indicating a dose response effect [44]. While our data suggest microbiome differences between FW and CW, these results are confounded by differing fructose concentrations, and more studies are needed to understand these relationships. It is unclear, moreover, whether these fructose-associated changes are primary or secondary dietary effects.

This was a study to investigate the effects of fructose on eating behavior and physiologic and metabolic parameters in pregnant rats and, as such, did not examine underlying mechanisms. The studies are limited by using a rodent model that may not fully represent human mechanisms of metabolic syndrome [45, 46]. We used commercially bred Sprague Dawley rats without selecting specific rat species to optimize dietary differences or human relevance [47–49]. Finally, the small sample size may have compromised detection of meaningful differences in some of the measured variables [50].

Using a pregnancy stress rat model, we conclude that ad libitum dietary fructose leads to increased consumption of fructose-containing food that results in increasing the carbohydrate dietary portion. When given a fructose-containing soft drink, this results in an unexpected alteration in dietary pattern characterized by an increase in fluid intake and urinary output. When a fructose-containing soft drink (CS) is substituted for water (CW), this is associated with decreased chow ingestion and consequent reduced fat and protein consumption. We also confirmed previous findings that a high-fructose chow diet during pregnancy is associated with hypertension and dyslipidemia not observed in CW or CS rats. The fructose dietary changes are associated with metabolic, physiological, and microbiome alterations. The relationships between high fructose and overall carbohydrate consumption are important nutritional considerations. Further research is needed to explore the mechanisms of these changes and their broader implications for pregnancy and long-term female health.

## Data Availability

The datasets used and/or analyzed during the current study are available from the corresponding author on reasonable request. The raw sequencing data was uploaded to the NCBI SRA. The accession to cite for the SRA data is PRJNA1161717. The data will be released on 2025-10-01.

## Supporting Information

Supporting Information Additional supporting information can be found online in the Supporting Information section. Table S1: Food nutritional label. Table S2: Data of CW, CS, and FW rats at prepregnancy (Pre), midpregnancy (Mid, Days 8–14), and late pregnancy (Late, Days 15–22). Table S3: Major organ weights and liver and kidney pathological gradings of CW, CS, and FW rats. Figure S1: Scatter plots of rat caloric and macronutrient intake during pregnancy. Figure S2: Total calories (A) and percent carbohydrate (B), fat (C), and protein (D) calories/total calories normalized to body weight of rats after 24-h metabolic cage monitoring. The insert in (B) shows the percent of carbohydrate calories from solid chow or liquid drink out of the total intake calories with CS diet. Figure S3: Scatter plots of rat serum metabolic parameters and systolic blood pressure during pregnancy. Figure S4: Representative images of rat liver and kidney histological sections from late pregnancy. Liver: hematoxylin and eosin (H&E) staining, 200X magnification; kidney: H&E and periodic acid-Schiff (PAS) staining, 100X magnification. One FW reference liver section shows signs of microsteatosis (black arrow), but no other liver histopathological changes (steatosis, ballooning, and fibrosis) nor kidney pathological changes (glomerulosclerosis, tubulointerstitial fibrosis, and inflammation) were detected in CW or CS.
