# Supplementary material for: Impact of Fructose-Enhanced Solid and Soft Drink Diets on Metabolism, Physiology, and Gut Microbiome in Pregnant Rats
Source: Biomed Res Int. 2025 Aug 13;2025:6902453. doi: 10.1155/bmri/6902453 (PMC12367382; doi:10.1155/bmri/6902453)
Supplement: Supporting Information — Additional supporting information can be found online in the Supporting Information section. Table S1: Food nutritional label. Table S2: Data of CW, CS, and FW rats at prepregnancy (Pre), midpregnancy (Mid, Days 8–14), and late pregnancy (Late, Days 15–22). Table S3: Major organ weights and liver and kidney pathological gradings of CW, CS, and FW rats. Figure S1: Scatter plots of rat caloric and macronutrient intake during pregnancy. Figure S2: Total calories (A) and percent carbohydrate (B), fat (C), and protein (D) calories/total calories normalized to body weight of rats after 24-h metabolic cage monitoring. The insert in (B) shows the percent of carbohydrate calories from solid chow or liquid drink out of the total intake calories with CS diet. Figure S3: Scatter plots of rat serum metabolic parameters and systolic blood pressure during pregnancy. Figure S4: Representative images of rat liver and kidney histological sections from late pregnancy. Liver: hematoxylin and eosin (H&E) staining, 200X magnification; kidney: H&E and periodic acid-Schiff (PAS) staining, 100X magnification. One FW reference liver section shows signs of microsteatosis (black arrow), but no other liver histopathological changes (steatosis, ballooning, and fibrosis) nor kidney pathological changes (glomerulosclerosis, tubulointerstitial fibrosis, and inflammation) were detected in CW or CS. [file 6902453.f1.doc]

TableS1 Food Nutritional Label.

| **Standard chow**  **(Teklad global 18% protein rodent diet)** | | | **60% fructose chow**  **(TD.89247)** | | | **Sprite® ^1^** | | | **Water** |
| --- | --- | --- | --- | --- | --- | --- | --- | --- | --- |
| **Calories** | **Unit** | **Amount** |  | **Unit** | **Amount** |  | **Unit** | **Amount** | **Among** |
| Total | Kcal/g | 3.1 |  | Kcal/g | 3.6 |  | Kcal/ml | 0.394 | 0 |
| from carbohydrate | % | 58 |  | % | 67 |  | % | 100 | 0 |
| from fat | % | 18 |  | % | 13 |  | % | 0 | 0 |
| from protein | % | 24 |  | % | 20 |  | % | 0 | 0 |
| **Ingredient** |  |  |  |  |  |  |  |  |  |
| **Macronutrients** |  |  |  |  |  |  |  |  |  |
| Crude Protein | % | 18.6 | Casein | % | 20.7 |  |  | 0 |  |
| Fat | % | 6.2 | Lard | % | 5 |  |  | 0 |  |
| Carbohydrate | % | 44.2 | Fructose | % | 60 | High fructose corn syrup | % (g per 100ml) | 10.7 |  |
| Crude Fiber | % | 3.5 | Cellulose | % | 8.0 |  |  | 0 |  |
| Neutral Detergent Fiber | % | 14.7 |  |  |  |  |  |  |  |
| Ash | % | 5.3 |  |  |  |  |  |  |  |
|  |  |  | Green Food Color | g/Kg | 0.15 |  |  |  |  |
| **Minerals** |  |  |  |  |  |  |  |  |  |
|  |  |  | **Mineral Mix, Rogers-Harper (170760)** | g/Kg | 50 |  |  |  |  |
| Calcium | % | 1 | Calcium carbonate | g/Kg | 14.6 |  |  | 0 |  |
| Phosphorus | % | 0.7 | Potassium phosphate, monobase | g/Kg | 17.2 |  |  |  |  |
| Non-phytate phosphorus | % | 0.4 | Calcium phosphate, dibasic, dihydrate | g/Kg | 0.22 |  |  |  |  |
| Sodium | % | 0.2 | Sodium chloride | g/Kg | 12.53 | Sodium | mg/L | 183 |  |
| Potassium | % | 0.6 |  |  |  |  |  | 0 |  |
| Chloride | % | 0.4 |  |  |  |  |  |  |  |
| Magnesium | % | 0.2 | Magnesium sulfate, monohydrate | g/Kg | 0.06 |  |  |  |  |
|  |  |  | Magnesium sulfate, heptahydrate | g/Kg | 4.99 |  |  |  |  |
| Zinc | mg/kg | 70 | Zinc chloride | g/Kg | 0.01 |  |  |  |  |
|  |  |  | Zinc Carbonate | g/Kg | 0.04 |  |  |  |  |
| Manganese | mg/kg | 100 |  |  |  |  |  |  |  |
| Copper | mg/kg | 15 | Cupric sulfate | g/Kg | 0.08 |  |  |  |  |
| Iodine | mg/kg | 6 | Potassium iodide | mg/Kg | 0.25 |  |  |  |  |
| Iron | mg/kg | 200 | Ferric citrate | g/Kg | 0.31 |  |  | 0 |  |
| Selenium | mg/kg | 0.23 | Sodium selenite | mg/Kg | 0.75 |  |  |  |  |
|  |  |  | Ammonium paramolybdate, tetrahydrate | mg/Kg | 1.15 |  |  |  |  |
| **Amino Acids** |  |  |  |  |  |  |  |  |  |
| Aspartic acid | % | 1.4 |  |  |  |  |  |  |  |
| Glutamic acid | % | 3.4 |  |  |  |  |  |  |  |
| Alanine | % | 1.1 |  |  |  |  |  |  |  |
| Glycine | % | 0.8 |  |  |  |  |  |  |  |
| Threonine | % | 0.7 |  |  |  |  |  |  |  |
| Proline | % | 1.6 |  |  |  |  |  |  |  |
| Serine | % | 1.1 |  |  |  |  |  |  |  |
| Leucine | % | 1.8 |  |  |  |  |  |  |  |
| Isoleucine | % | 0.8 |  |  |  |  |  |  |  |
| Valine | % | 0.9 |  |  |  |  |  |  |  |
| Phenylalnine | % | 1 |  |  |  |  |  |  |  |
| Tyrosine | % | 0.6 |  |  |  |  |  |  |  |
| Methionine | % | 0.4 | DL-Methionine | g/Kg | 3 |  |  |  |  |
| Cystine | % | 0.3 |  |  |  |  |  |  |  |
| Lysine | % | 0.9 |  |  |  |  |  |  |  |
| Histidine | % | 0.4 |  |  |  |  |  |  |  |
| Arginine | % | 1 |  |  |  |  |  |  |  |
| Tryptophan | % | 0.2 |  |  |  |  |  |  |  |
| **Vitamins** |  |  |  |  |  |  |  |  |  |
|  |  |  | **Vitamin Mix, Teklad (40060)** | g/Kg | 10 |  |  |  |  |
| Vitamin A | IU/g | 15 | Vitamin A Palmitate | IU/g | 19.8 |  |  |  |  |
| Vitamin D3 | IU/g | 1.5 | Vitamin D3, cholecalciferol | IU/g | 2.2 |  |  | 0 |  |
| Vitamin E | IU/kg | 110 | Vitamin E, DL-alpha tocopheryl acetate | IU/Kg | 121.1 |  |  |  |  |
| Vitamin K3 (menadione) | mg/kg | 50 | Vitamin K3, menadione | mg/Kg | 50 |  |  |  |  |
| Vitamin B1 (thiamin) | mg/kg | 17 | Thiamin (81%) | mg/Kg | 22 |  |  |  |  |
| Vitamin B2 (riboflavin) | mg/kg | 15 | Riboflavin | mg/Kg | 22 |  |  |  |  |
| Niacin (nicotinic acid) | mg/kg | 70 | Niacin | mg/Kg | 99 |  |  |  |  |
| Vitamin B6 (pyridoxine) | mg/kg | 18 | Pyridoxine HCl | mg/Kg | 22 |  |  |  |  |
| Pantothenic Acid | mg/kg | 33 | Calcium Pantothenate | mg/Kg | 66 |  |  |  |  |
| Vitamin B12 (cyanocobalamin) | mg/kg | 0.08 | Vitamin B12 (0.1% in mannitol) | mg/Kg | 30 |  |  |  |  |
| Biotin | mg/kg | 0.4 | Biotin | mg/Kg | 0.441 |  |  |  |  |
| Folate | mg/kg | 4 | Folic Acid | mg/Kg | 1.982 |  |  |  |  |
| Choline | mg/kg | 1200 | Choline Dihydrogen Citrate | mg/Kg | 3497 |  |  |  |  |
|  |  |  | Vitamin C, ascorbic acid, coated (97.5%) | g/Kg | 1.017 |  |  |  |  |
|  |  |  | p-Aminobenzoic Acid (Vitamin B10) | g/Kg | 0.110 |  |  |  |  |
|  |  |  | Inositol | g/Kg | 0.110 |  |  |  |  |
|  |  |  | Corn Starch | g/Kg | 4.667 |  |  |  |  |
| **Fatty Acids** |  |  |  |  |  |  |  |  |  |
| C16:0 Palmitic | % | 0.7 |  |  |  |  |  |  |  |
| C18:0 Stearic | % | 0.2 |  |  |  |  |  |  |  |
| C18:1ω9 Oleic | % | 1.2 |  |  |  |  |  |  |  |
| C18:2ω6 Linoleic | % | 3.1 |  |  |  |  |  |  |  |
| C18:3ω3 Linolenic | % | 0.3 |  |  |  |  |  |  |  |
| Total Saturated | % | 0.9 |  |  |  |  |  |  |  |
| Total Monounsaturated | % | 1.3 |  |  |  |  |  |  |  |
| Total Polyunsaturated | % | 3.4 |  |  |  |  |  |  |  |
| **Other** |  |  |  |  |  |  |  |  |  |
| Cholesterol | mg/kg | - |  |  |  |  |  | 0 |  |

Note: ^1^Ingredients of Sprite®: Carbonated water, high fructose corn syrup, citric acid, natural flavors, sodium citrate, sodium benzoate (to protect taste). (<https://www.sprite.com/products>)

Table S2 Data of CW, CS and FW Rats at Pre-pregnancy (Pre), Mid-pregnancy (Mid, Day 8-14), and Late Pregnancy (Late, Day15-22).

|  |  | **CW (n=5)** |  |  | | **CS (n=5)** |  |  | **FW (n=5)** |  |
| --- | --- | --- | --- | --- | --- | --- | --- | --- | --- | --- |
|  | Pre | Mid | Late | Pre | Mid | | Late | Pre | Mid | Late |
| Body weight  (BW, g) | 217.3±10.8 | 244.7±25.4* | 314.62±16.2*^ | 206.5±11.6 | 249.6±19.0* | | 298.5±30.4*^ | 203.3±6.8 | 241.0±16.0* | 297.9±33.1*^ |
| Chow/BW | 0.097±0.017 | 0.101±0.033 | 0.088±0.012 | 0.093±0.018 | 0.077±0.009 | | 0.074±0.002 | 0.085±0.008 | 0.108±0.032b | 0.062±0.019^ |
| Feces/BW | 0.021±0.005 | 0.026±0.007 | 0.028±0.005 | 0.025±0.009 | 0.018±0.009 | | 0.022±0.012 | 0.022±0.011 | 0.014±0.006 | 0.011±0.003b* |
| Liquid/BW  (ml/g) | 0.140±0.043 | 0.141±0.041 | 0.148±0.011 | 0.178±0.079 | **0.284±0.111a*** | | 0.212±0.050 | 0.168±0.084 | 0.152±0.042b | 0.152±0.096 |
| Urine/BW  (ml/g) | 0.084±0.042 | 0.086±0.037 | 0.094±0.056 | 0.099±0.080 | **0.197±0.086a*** | | 0.134±0.052^ | 0.095±0.065 | 0.120±0.047 | 0.074±0.029 |
| Total calories/BW  (kcal/g) | 0.301±0.051 | 0.313±0.101 | 0.271±0.036 | 0.289±0.055 | 0.352±0.032 | | 0.312±0.018 | 0.263±0.025 | 0.391±0.117* | 0.224±0.068^ |
| % of calories  from carbohydrates | 58.0±0.0 | 58.0±0.0 | 58.0±0.0 | 58.0±0.0 | **71.1±4.5a*** | | **69.2±2.1a*** | 58.0±0.0 | 67.0±0.0ab* | 67.0±0.0a* |
| % of calories from fat | 18.0±0.0 | 18.0±0.0 | 18.0±0.0 | 18.0±0.0 | 12.4±1.9a* | | 13.2±0.9a* | 18.0±0.0 | 13.0±0.0a* | 13.0±0.0a* |
| % of calories from protein | 24.0±0.0 | 24.0±0.0 | 24.0±0.0 | 24.0±0.0 | 16.5±2.5a* | | 17.6±1.2a* | 24.0±0.0 | 20.0±0.0ab* | 20.0±0.0ab* |
| Triglyceride  (mg/dl) | 32.3±20.1 | 27.8±10.1 | 516.9±189.2*^ | 29.4±15.2 | 103.9±45.4 | | 548.6±84.6*^ | 21.7±8.9 | 121.5±87.0 | 496.9±261.6*^ |
| LDL/VLDL  (mg/dl) | 30.3±9.8 | 23.8±7.0 | 50.1±18.2^ | 31.1±7.7 | 20.3±8.2 | | 54.5±15.7*^ | 28.3±8.7 | 18.5±9.4 | **82.3±37.4ab*^** |
| Total cholesterol  (mg/dl) | 87.2±35.2 | 67.2±9.4 | 80.7±23.0 | 80.3±25.3 | 60.9±14.5 | | 87.6±23.8 | 82.7±23.0 | 55.3±24.2 | 95.4±28.6^ |
| Blood Pressure  (mmHg) | 122.5±16.4 | 128.2±10.2 | 124.4±16.3 | 113.3±10.9 | 129.8±7.4 | | 130.8±11.0 | 124.9±8.3 | 148.2±13.6* | 104.2±18.3ab*^ |
| Creatinine  (mg/dl) | 0.62±0.23 | 0.36±0.05 | 0.40±0.04 | 0.60±0.32 | 0.49±0.05 | | 0.40±0.20 | 0.58±0.49 | 0.07±0.04 | 0.19±0.20 |

Note: Data were analyzed by two-way ANOVA (considering one independent factor of diet and with one independent repeated-measures factor of time), following Tukey *post hoc* test. P<0.05 was indicated by * (vs Pre in the respective dietary group), ^ (vs Mid in the respective dietary group), a (vs CW at the respective timepoint), or b (vs CS at the respective timepoint). Eleven measurements in serum creatinine data were below the detection limit(0.1mg/dl) and replaced with 0.05mg/dl (half of the detection limit). Serum creatinine data were analyzed using multiple non-parametric comparisons followed by the Bonferroni-Dunn correction method. The comparisons between two dietary groups at one timepoint were analyzed by Mann-Whitney tests. The comparisons between two timepoints in one dietary group were analyzed by Wilcoxon matched-pairs signed rank tests.

Table S3 Major Organ Weights and Liver and Kidney Pathological Gradings of CW, CS, FW Rats

|  | CW (n=5) | CS (n=5) | FW (n=5) |
| --- | --- | --- | --- |
| Liver (g) | 10.922±1.717 | 13.114±0.257 | 11.408±1.945 |
| Left kidney (g) | 0.869±0.102 | 0.842±0.097 | 0.912±0.154 |
| Right kidney (g) | 0.833±0.071 | 0.784±0.062 | 0.855±0.157 |
| Heart (g) | 1.072±0.225 | 1.145±0.076 | 0.972±0.053 |
| Retroperitoneal fat (g) | 2.645±0.898 | 4.804±0.716 | 4.562±2.581 |
| Bladder (g) | 0.144±0.044 | 0.172±0.033 | 0.131±0.028 |
| Pancreas (g) | 0.943±0.448 | 1.208±0.409 | 1.103±0.285 |
| Baby number | 12.4±2.3 | 12.8±1.3 | 9.2±5.5 |
| Baby weight(g) | 3.66±1.53 | 4.53±1.68 | 3.75±2.09 |
| **Hepatic pathological evaluation** |  |  |  |
| Steatosis severity | 0 | 0 | 0.2±0.4 |
| Type of steatosis |  |  |  |
| Micro- | None | None | Yes (1 out of 5) |
| Macro- | None | None | None |
| Inflammation | 0 | 0 | 0 |
| Ballooning | 0 | 0 | 0 |
| Fibrosis | 0 | 0 | 0 |
| **Renal pathological evaluation** |  |  |  |
| Tubular injury | 0.2±0.4 | 0 | 0 |
| %Sclerosis glomeruli | 0 | 0 | 0 |
| Interstitial inflammation | 0 | 0 | 0 |
| Interstitial fibrosis | 0 | 0 | 0 |
| **Renal computer-assisted imaging quantification** |  |  |  |
| Glomerular volume (X 10^6^ μm^3^) | 0.733±0.115 | 0.907±0.144 | 0.814±0.163 |
| Tubular diameter (μm) | 35.8±3.88 | 36.9±2.34 | 39.6±4.22 |


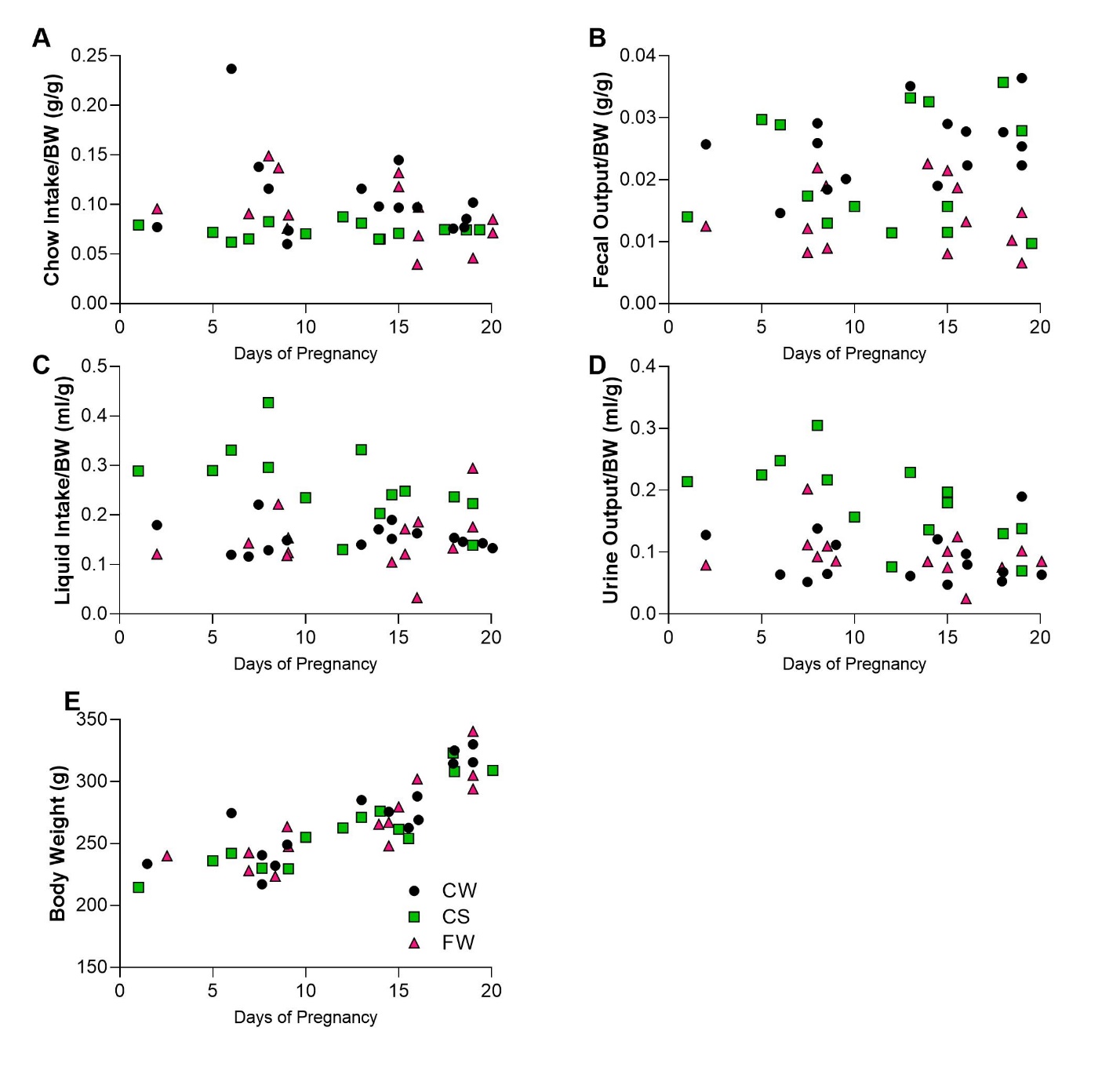


Figure S1 Scatter Plots Showing Rat Caloric and Macronutrient Intake during Pregnancy


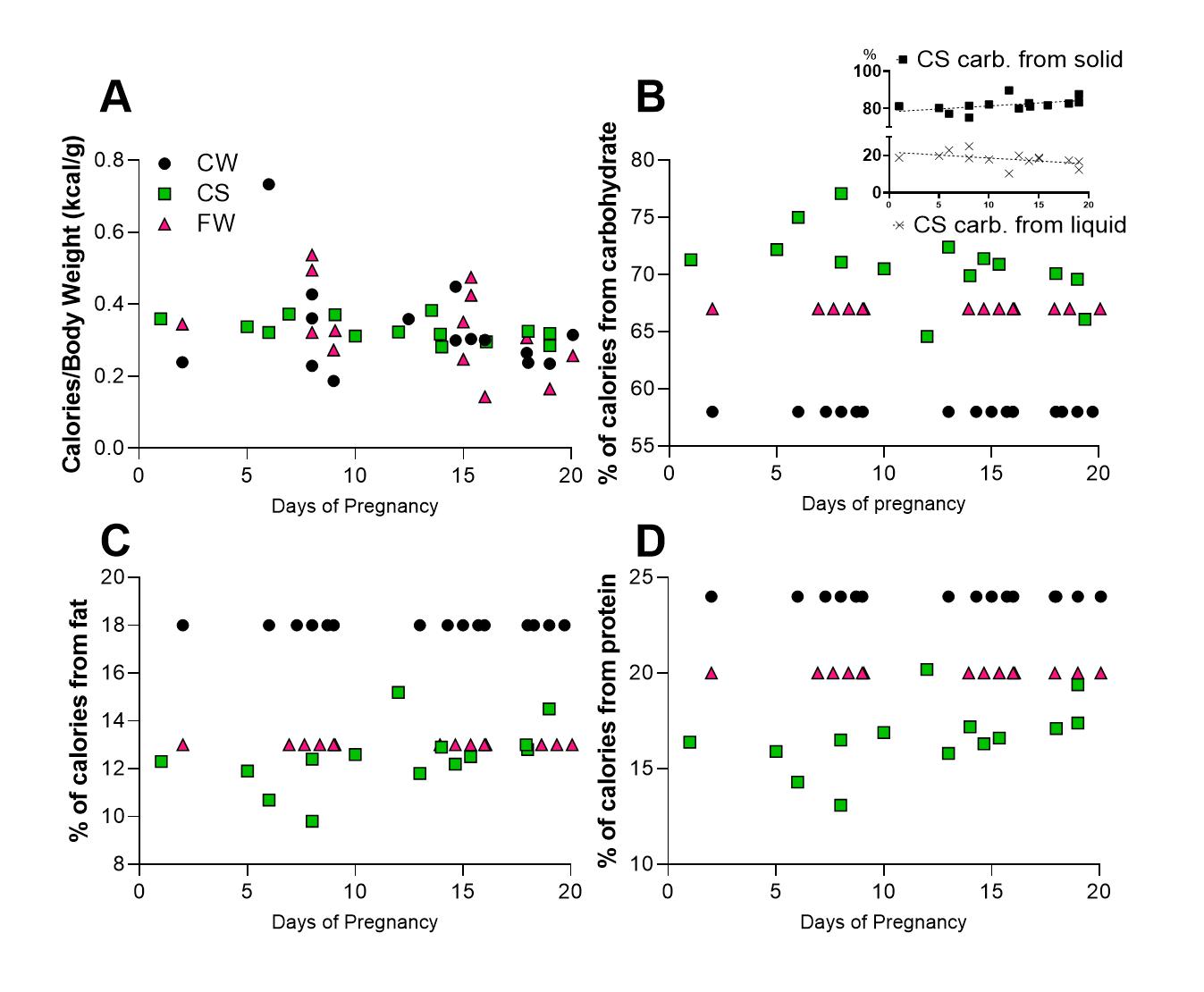


Figure S2 Total Calories(A) and Percent Carbohydrate (B), Fat (C), and Protein (D) Calories/Total Calories Normalized to Body Weight of Rats after 24-Hr Metabolic Cage Monitoring. The insert in B shows the percent of carbohydrate calories from solid chow or liquid drink out of the total intake calories in rats with CS diet.


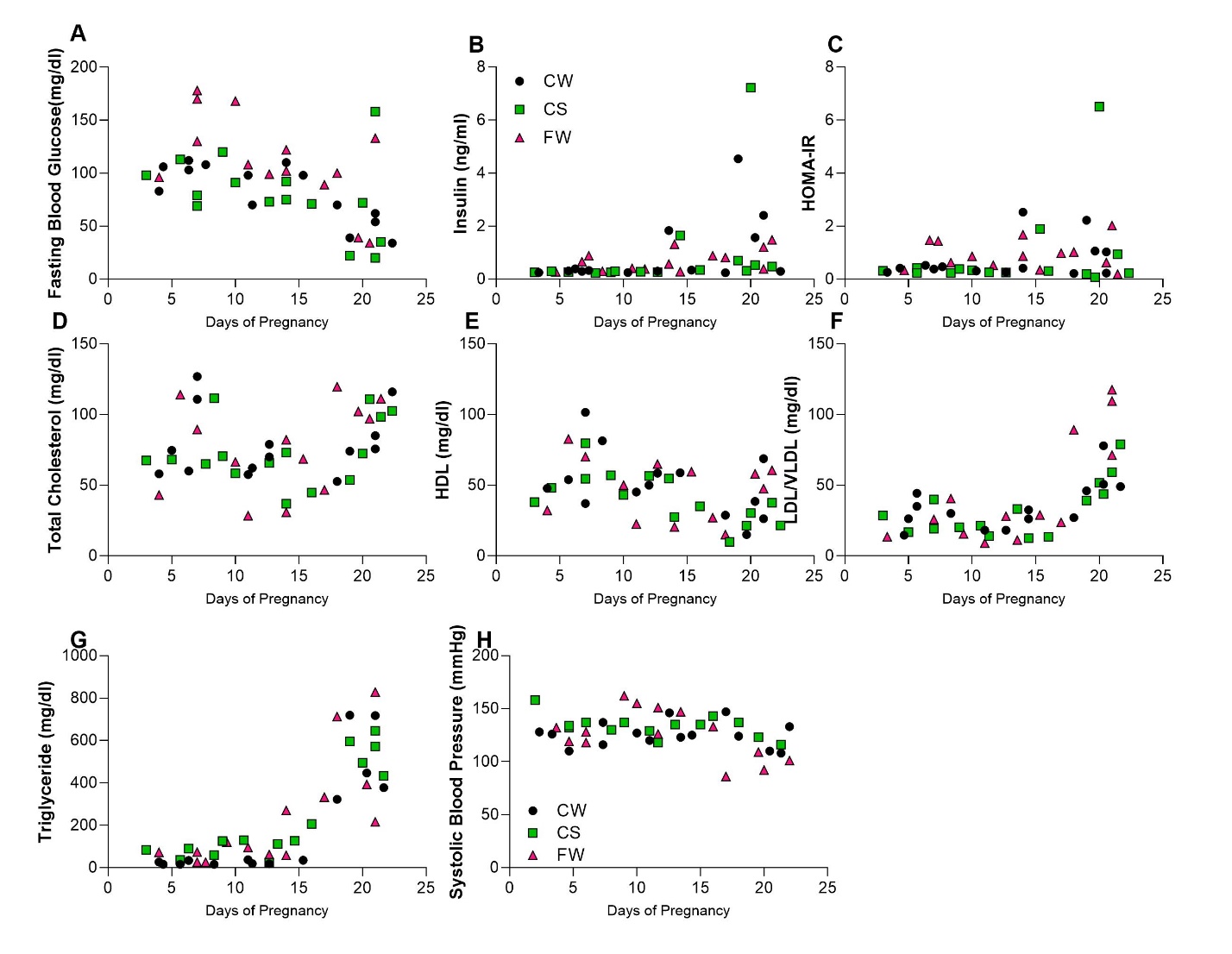


Figure S3 Scatter Plots of Rat Serum Metabolic Parameters and Systolic Blood Pressure during Pregnancy


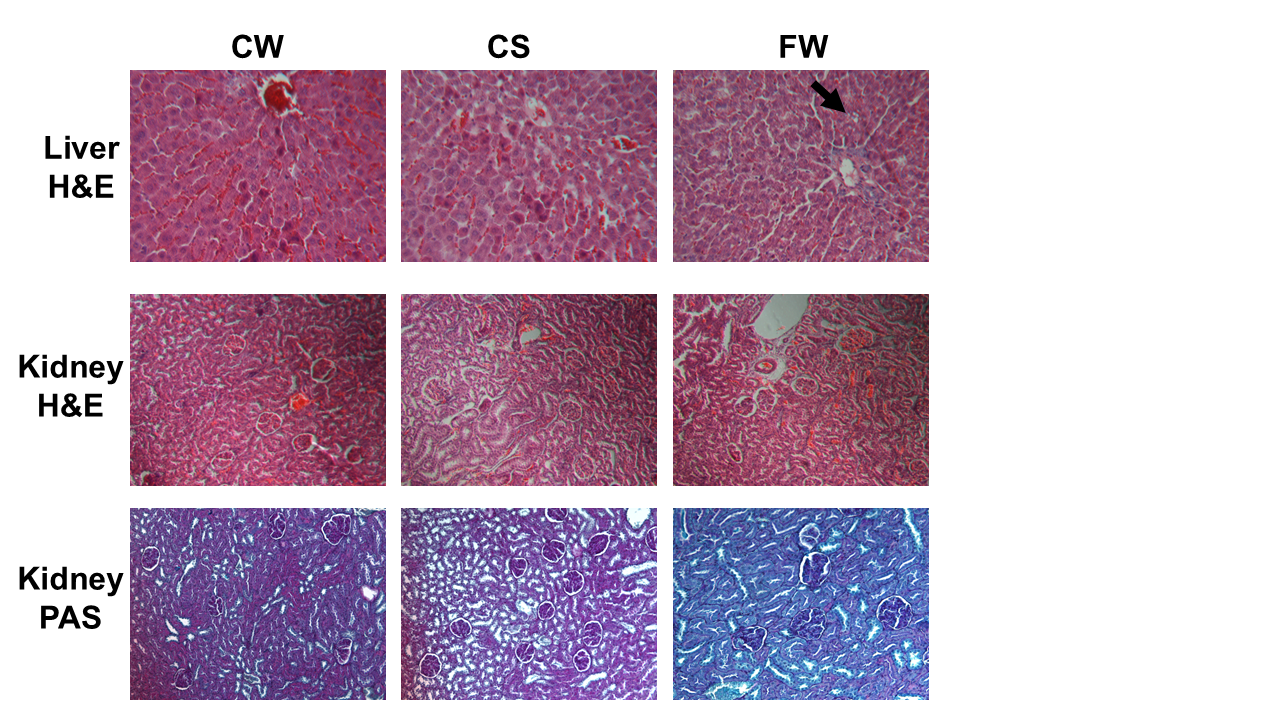


Figure S4 Representative Images of Rat Liver and Kidney Histological sections From Late Pregnancy. Liver: Hematoxylin and Eosin(H&E) Staining, 200X Magnification; Kidney: H&E And Periodic Acid-Schiff (PAS) Staining, 100X Magnification One FW reference liver section shows signs of microsteatosis (black arrow), but no other liver histopathological changes (steatosis, ballooning, and fibrosis) nor kidney pathological changes (glomerulosclerosis, tubulointerstitial fibrosis, and inflammation) were detected in CW or CS.
